# Draft genome sequence of non-shiga toxin-producing *Escherichia coli* O157 NCCP15738

**DOI:** 10.1186/s13099-016-0096-2

**Published:** 2016-04-19

**Authors:** Taesoo Kwon, Jung-Beom Kim, Young-Seok Bak, Young-Bin Yu, Ki Sung Kwon, Won Kim, Seung-Hak Cho

**Affiliations:** School of Biological Sciences, Seoul National University, 1 Gwanak-ro, Gwanak-gu, Seoul, 151-742 Republic of Korea; Division of Biosafety Evaluation and Control, Korea National Institute of Health, Cheongju, 363-951 Republic of Korea; Department of Food Science and Technology, Sunchon National University, Sunchon, Jeonnam 540-950 Republic of Korea; Department of Emergency Medical Service, College of Medical Science, Konyang University, Daejeon, 302-832 Republic of Korea; Department of Biomedical Laboratory Science, College of Medical Science, Konyang University, Daejeon, 302-832 Republic of Korea; New Hazardous Substances Team, National Institute of Food and Drug Safety Evaluation, Cheongju, 363-700 Republic of Korea; Division of Enteric Diseases, Center for Infectious Diseases, Korea National Institute of Health, Cheongju, 363-951 Republic of Korea

**Keywords:** Non-shiga toxin-producing *Escherichia coli* O157, Draft genome, Dual flagella

## Abstract

**Background:**

The non-shiga toxin-producing *Escherichia coli* (non-STEC) O157 is a pathogenic strain that cause diarrhea but does not cause hemolytic-uremic syndrome, or hemorrhagic colitis. Here, we present the 5-Mb draft genome sequence of non-STEC O157 NCCP15738, which was isolated from the feces of a Korean patient with diarrhea, and describe its features and the structural basis for its genome evolution.

**Results:**

A total of 565-Mbp paired-end reads were generated using the Illumina-HiSeq 2000 platform. The reads were assembled into 135 scaffolds throughout the de novo assembly. The assembled genome size of NCCP15738 was 5,005,278 bp with an N50 value of 142,450 bp and 50.65 % G+C content. Using Rapid Annotation using Subsystem Technology analysis, we predicted 4780 ORFs and 31 RNA genes. The evolutionary tree was inferred from multiple sequence alignment of 45 *E. coli* species. The most closely related neighbor of NCCP15738 indicated by whole-genome phylogeny was *E. coli* UMNK88, but that indicated by multilocus sequence analysis was *E. coli* DH1(ME8569).

**Conclusions:**

A comparison between the NCCP15738 genome and those of reference strains, *E. coli* K-12 substr. MG1655 and EHEC O157:H7 EDL933 by bioinformatics analyses revealed unique genes in NCCP15738 associated with lysis protein S, two-component signal transduction system, conjugation, the flagellum, nucleotide-binding proteins, and metal-ion binding proteins. Notably, NCCP15738 has a dual flagella system like that in *Vibrio parahaemolyticus*, *Aeromonas* spp., and *Rhodospirillum centenum*. The draft genome sequence and the results of bioinformatics analysis of NCCP15738 provide the basis for understanding the genomic evolution of this strain.

**Electronic supplementary material:**

The online version of this article (doi:10.1186/s13099-016-0096-2) contains supplementary material, which is available to authorized users.

## Background

*Escherichia coli* is a gram-negative bacterium that colonizes the human gastrointestinal tract. Most *E. coli* serotypes are non-pathogenic but some serotypes cause food poisoning. *E. coli* strains are divided into three subgroups, according to their pathogenicity: nonpathogenic, pathogenic, and extra intestinal pathogenic *E. coli*. There are 190 serotypes [1] of *E. coli*, based on the major surface antigens (O, H, and K) [2]. The serotype O157:H7 is the major strain in the enterohemorrhagic *E. coli* (EHEC) serotype and since 1982, these strains have been found to be important food-borne pathogens [3]. This type of *E. coli* can cause hemorrhagic colitis and hemolytic uremic syndrome (HUS) [4, 5]. O157:H7 can be identified by a combination of biochemical and immunological markers, such as sorbitol [6] in combination with O antigen [7]. *E. coli* O157:H7 is characterized by the expression of shiga-like toxins even though it produces various other virulence factors [8–10]. Shiga toxins are classified into two major groups, Stx1 and Stx2, which are encoded on a prophage [11]. These genes can be transferred horizontally to *E. coli* and other *Enterobacteriaceae* species [12], allowing transformation of shiga-like toxin non-producing strains into shiga-like toxin-producing strains [13]. The capillary endothelial cells are the major target sites of shiga toxins released by shiga toxin-producing *E. coli* (STEC). Specifically, the shiga toxins target the globotriaosylceramide receptor on the cells and are transported into the cells by receptor-mediated endocytosis [14]. Shiga toxins halt protein synthesis by cleaving an adenine base from the ribosomes of the intruded cells [15]. This blockage can cause kidney failure, as in HUS [14].

Although serotype STEC O157 strains are prevalent, non-STEC O157 strains have also been reported in children with diarrhea [16]. Very little is known about the symbiosis and pathogenicity of non-STEC O157 strains in the host; therefore, their genomes should be sequenced to assess horizontal gene transfer (HGT) and to understand the evolution of these strains. In this study, we performed genomic sequencing to investigate the genetic background of the evolution of non-STEC O157 NCCP15738 isolated from a patient with diarrhea. We also performed genomic comparison between the genomes of NCCP15738 and two reference strains, *E. coli* K-12 substr. MG1655 [17] and EHEC O157:H7 str. EDL933 [18] to study their evolution and phylogenetic linkage.

## Methods

### Strain, isolation, and serotyping

A fecal sample from a patient with diarrhea was plated on MacConkey agar directly or, occasionally, after enrichment in trypticase soy broth containing vancomycin (Sigma Chemicals Co., St. Louis, MO). Candidate colonies were then plated on trypticase soy agar medium and biochemically characterized using the API20E system (Biomerieux, Marcy l’Etoile, France). For O-antigen determination, we used the method described by Guinee et al. [7] and all available O (O1–O181) antisera. All antisera were absorbed with the corresponding cross-reacting antigens to remove non-specific agglutinins. The O antisera were produced at Laboratorio de Referencia de *E. coli* (Lugo, Spain [http://www.lugo.usc.es/ecoli]). This research was approved by the Research Ethics Committee of the Korea Centers for Disease Control and Prevention, and written informed consent was obtained from the patient. The isolated strain was deposited at the National Culture Collection for Pathogens (NCCP) at Korea National Institute of Health under the accession number NCCP15738. *E. coli* K-12 substr. MG1655 and EHEC O157:H7 str. EDL933 were used as the reference strains because these strains represent non-STECs and STECs, respectively.

### Library preparation and whole-genome sequencing

The genomic DNA of NCCP15738 was purified and fragmented randomly. After fragmentation, the overhangs were converted into blunt ends using T4 DNA polymerase, Klenow Fragment, and T4 Polynucleotide Kinase (New England Biolabs, MA, USA). Sequencing adapters were ligated to the ends of the end-repaired DNA fragments. The DNA fragments that met the required length were retained by gel electrophoresis and amplified by PCR. We used the Illumina-HiSeq 2000 (Illumina, San Diego, CA, USA) platform for whole-genome sequencing and produced 565,810,000 bp data with paired end reads of 90-bp length and 500-bp insert size.

### Genome assembly and annotation

For quality control of the sequencing data, the following steps were employed. First, reads with more than 9 % Ns’ bases or low complexity reads were discarded. Second, reads with more than 40 bases of low quality (≤Q20) were discarded. Third, adapter sequences with at least 15 bp overlap between adapter and reads that allowed 3 bp mismatches were removed. Fourth, duplicated reads were discarded. After quality control removals, we obtained 504 Mbp of high quality reads. SOAPdenovo (version 1.05) [19] was used for de novo assembly of the genome using the high quality reads. For the purpose of assembly correction, all reads that passed the quality control were aligned against the assembly result using SOAPaligner (version 2.21) [20]. The single base error of the assembly result was corrected using mapping information. Scaffolds over 500 bp in length were considered for downstream analysis. To predict open reading frames (ORFs) and annotate the ORFs, we used the RAST (Rapid Annotation using Subsystem Technology, version 4.0) [21] server pipeline. We compared the predicted CDSs (coding DNA sequences) of NCCP15738 to those of two *E. coli* strains, K-12 substr. MG1655 and *E. coli* O157:H7 str. EDL933, using OrthoMCL software (version 2.0.9) [22]. Orthologous protein sequences were clustered into groups and the orthologous proteins of all three *E. coli* strains in each group were counted. To identify the virulence factor genes in NCCP15738, we performed a BLAST (Basic Local Alignment Search Tool) search of whole NCCP15738 ORFs against the virulence factor genes listed in VFDB [23] with an e-value of 1e-5. Insertion sequences (ISs) were identified by mapping to a sequence database that was downloaded from IS Finder DB (http://www-is.biotoul.fr), using RepeatMasker (version 4.0.1) (http://www.repeatmasker.org). Phage-associated gene clusters in the scaffolds of NCCP15738 were searched using the PHAST server [24] (data not shown).

### Phylogenetic analysis and comparative genomic analysis

To infer the evolutionary history of NCCP15738, we performed a multiple sequence alignment of the whole genome using Mugsy (version 1.2.3) [25] and approximately-maximum-likelihood phylogenetic trees were inferred using FastTree (version 2.1.7) [26] with a GTR (generalized time-reversible) + CAT model [27]. The tree was visualized using FigTree (version 1.3.1) (http://tree.bio.ed.ac.uk/software/figtree/). In order to exclude the effect of HGT in our phylogenetic analysis, we used the multilocus sequence analysis (MLSA) method [28, 29]. Seven housekeeping genes (*adk, fumC, gyrB, icd, mdh, purA,* and *recA)* from 45 *E. coli* strains were retrieved and concatenated. A phylogenetic tree of multi locus sequence typing (MLST) genes was created using the method employed for whole-genome phylogenetic analysis. Mauve (version 2.3.1) [30] was used for comparative genomics using the Move Contig tool. The scaffolds were reordered against the complete genome of the reference *E. coli* strain, K-12 substr. MG1655. From the comparative genomic study, we identified a syntenic region that aligned against the reference genome. Unaligned scaffolds against the reference genome were defined as unique regions of NCCP15738. We also used the progressive alignment algorithm of Mauve for comparative alignment of NCCP15738, *E. coli* strain K-12 substr. MG1655, and *E. coli* strain O157:H7 str. EDL933 genomes. The BLAST algorithm was used to identify syntenic genes between the species and to analyze the genes of interest.

### Quality assurance

The genomic DNA was purified from a pure culture of a single bacterial isolate of NCCP15738. Potential contamination of the genomic library by other microorganisms was assessed using a BLAST search against the non-redundant database. We also checked for contamination by other genomes by confirming coverage distribution.

## Results and discussion

### General features

**Fig. 1:**
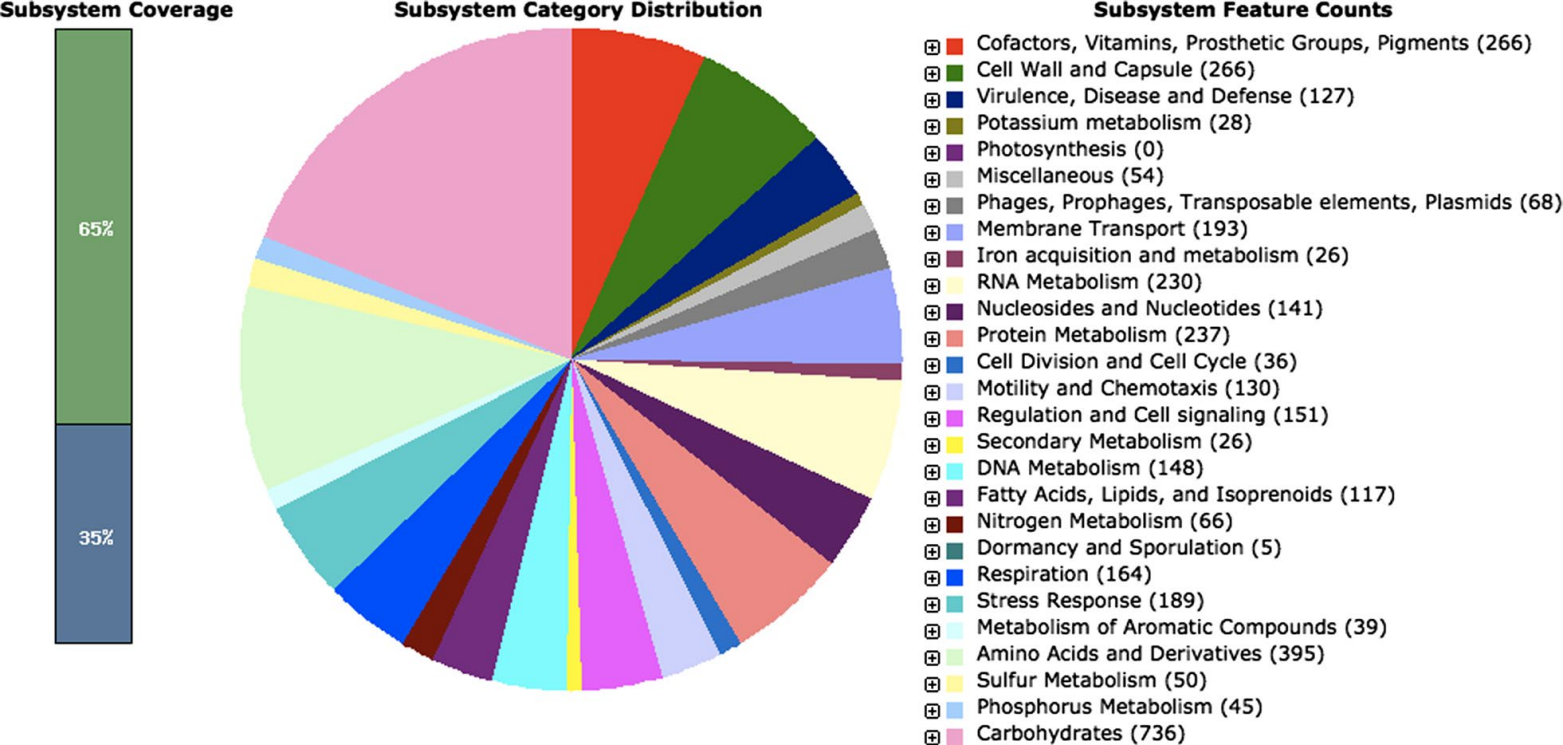
Subsystem category distribution of NCCP15738 based on SEED databases

Whole-genome sequencing by Illumina-HiSeq 2000 showed 565,810,000 bp with paired end reads that were 90 bp in length. After quality control, 504 Mbp of high quality reads were kept for assembly. The average sequencing depth was 86.7-fold coverage and the coverage ratio was 84.77 %. The high quality reads were assembled into 135 scaffolds by de novo assembly with an N50 value of 142,450 bp. The predicted genome size of NCCP15738 was 5,005,278 bp with 50.65 % G+C content. RAST analysis identified 4780 putative ORFs and 31 RNA genes, of which 4181 (80.6 %) could be functionally annotated (Fig. 1). The monosaccharides (212 ORFs) and central carbohydrate metabolism (135 ORFs) subsystems were significantly abundant among the subsystems (18.7 %). According to the subsystem results, we can assume that the NCCP15738 developed systems that can utilize various monosaccharides in addition to glucose to adapt to an extreme environment. A large number of ORFs were also associated with the “Amino acids and derivatives” subsystem (395 ORFs), “cofactors, vitamins, prosthetic groups, pigments” subsystem (266 ORFs) and “cell wall and capsule” subsystem (266 ORFs).

### Comparative genomics of NCCP15738 with other *E. coli* strains

**Fig. 2:**
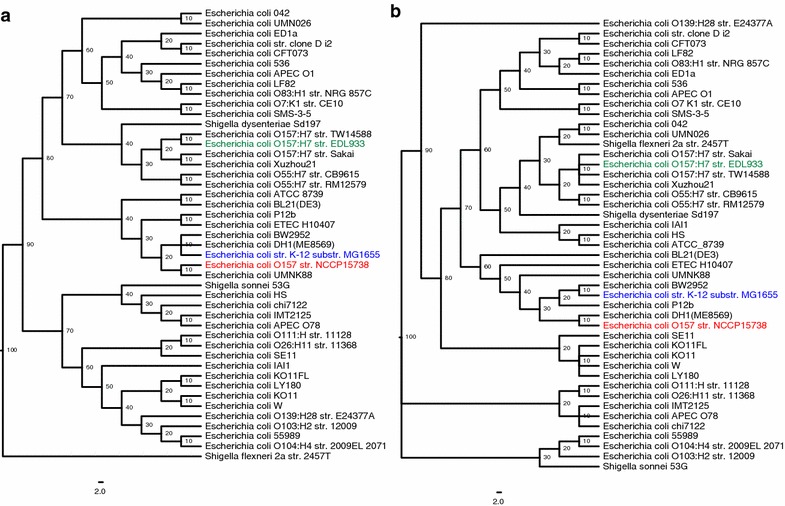
Phylogenetic tree of NCCP15738. **a** Whole-genome phylogeny, **b** MLSA phylogeny. Evolutionary time is scaled by 100; lower values imply relatively recent branching. The scale indicates the number of substitutions per site. NCCP15738 (*red*) was not placed in a single clade with *E. coli* K-12 substr. MG1655 (*blue*) in either the whole-genome phylogeny or the MLSA phylogeny. In addition, NCCP15738 did not belong to the *E. coli* O157:H7 serotype and was evolutionarily far from *E. coli* O157:H7 str. EDL933 (*green*). The most closely related neighbor indicated by whole-genome phylogeny was *E. coli* UMNK88, but that indicated by MLSA phylogeny was *E. coli* DH1 (ME8569)

The phylogenetic comparison of gene candidates predicted by SEED [31] revealed *E. coli* O104:H4 GOS1 [32] as the closest neighbor of NCCP15738 (score 513). To investigate the detailed evolutionary history of NCCP15738, we performed a multiple sequence alignment of 45 *E. coli* species including NCCP15738 (Fig. 2, Additional file 1: Table S1). Whole-genome phylogenetic analysis revealed that NCCP15738 did not cluster with *E. coli* strain K-12 substr. MG1655 into a single clade. Moreover, NCCP15738 was not grouped with *E. coli* O157:H7 str. EDL933. The most closely related neighbor of NCCP15738 was the pathogenic *E. coli* UMNK88 [33]. In MLSA, NCCP15738 clustered with *E. coli* DH1 (ME8569) into a single clade. The *E. coli* UMNK88 strain and K-12 substr. MG1655 were farther from NCCP15738 in the MLSA tree than in the whole-genome phylogenetic tree. However, this difference between the whole-genome phylogenetic tree and the MLST phylogenetic tree was not significant, as there was consensus in the topology among trees. It is concordant with previous research with Phylomark [34].

### Comparison of functional genes

**Fig. 3:**
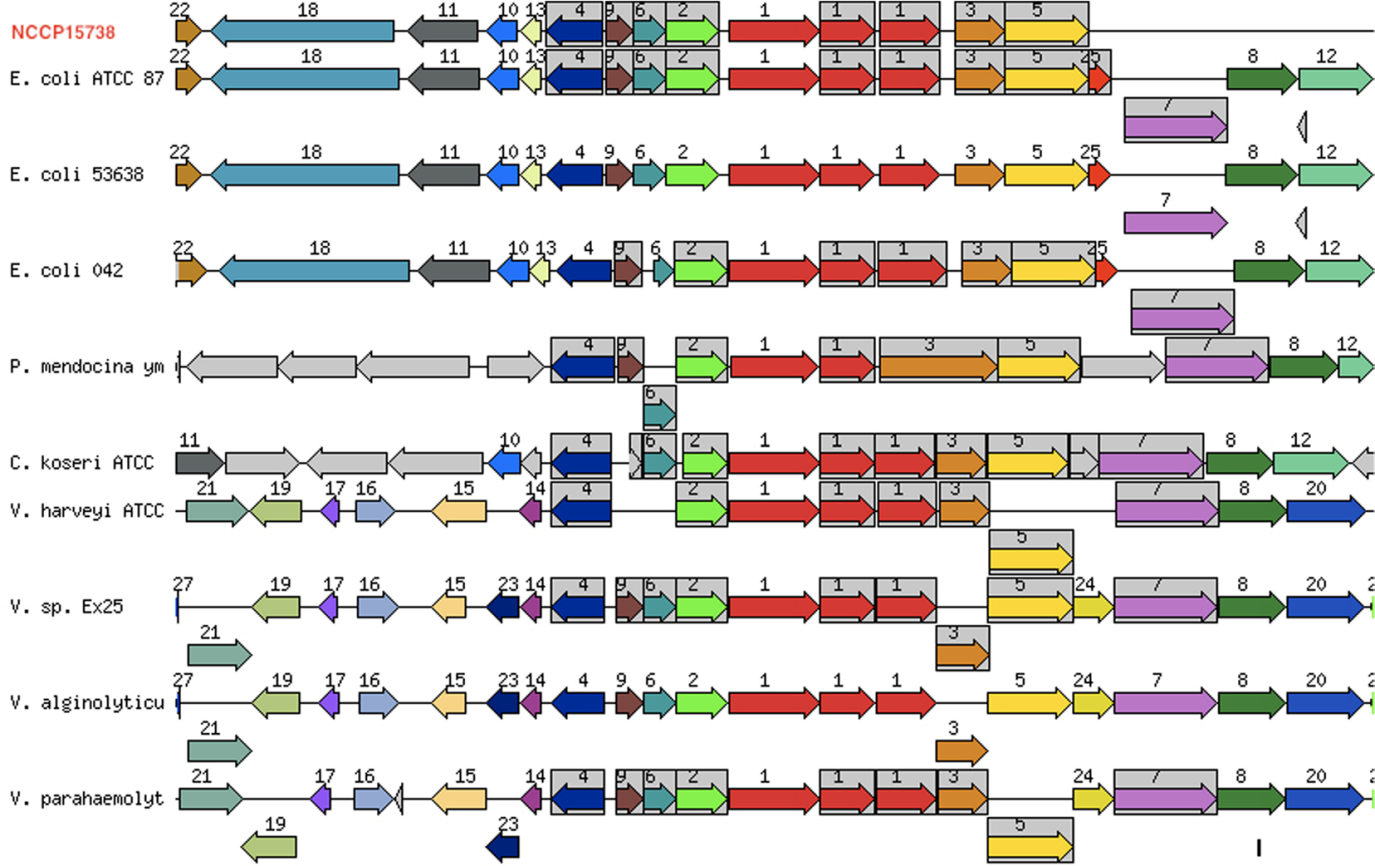
Comparative map of lateral flagella in NCCP15738 genome and other closely related species. Nine genes were highly conserved in 11 strains, but only seven genes (*1*–*6*, and *9*) were conserved in NCCP15738. Numbers indicate genes encoding for the following proteins: flagellar hook protein FlgE, flagellar basal-body rod protein FlgF and FlgG from *left to right* (*1*); FlgD (*2*); FlgH (*3*); FlgA (*4*); FlgI (*5*); FlgC (*6*); FlgB (*7*); FlgK (*8*); FlgL (*9*); hypothetical protein (10); hypothetical protein (11); lysine-N-methylase (EC 2.1.1.-) (*12*); hypothetical protein (*13*); LfgM (*14*); membrane-bound lytic murein transglycosylase D precursor (EC 3.2.1.-) (*15*); MutT/nudix family protein (*16*); hemolysin (*17*); Cps2A (*18*); hypothetical protein (*19*); putative flagellin (*20*); hypothetical protein (*21*); glycerol-3-phosphate cytidylyltransferase (EC 2.7.7.39) (*22*); LfgN (*23*); FlgJ (*24* and *25*). *Gray background boxes* indicate that the genes in the relative position are conserved in at least four species. The comparative map was created with the genome browser of the SEED viewer (version 2.0)

A comparison of NCCP15738 genes and those of the two reference strains, *E. coli* K-12 substr. MG1655 and *E. coli* O157:H7 str. EDL933 showed that most of the functional genes of NCCP15738 were conserved in the two reference strains, but 941 genes were unique (Additional file 2: Table S2). Unique genes in NCCP15738 included those encoding lysis protein S, a two-component signal transduction system, conjugation, the flagellum, nucleotide-binding proteins, and metal ion binding proteins, explain the phenotypic differences that result from environmental adaptation. In particular, NCCP15738 has a dual flagella system used for swarming in viscous media. This system resembles those found in *Vibrio parahaemolyticus*, *Aeromonas* spp., and *Rhodospirillum centenum* [35]. Sixty-five genes encoded the flagellar biosynthesis protein or the flagellar structural protein. Seven of the flagella-related proteins (1–6, and 9) were highly conserved in *V. parahaemolyticus* and in nine other strains (Fig. 3, Additional file 3: Table S3). Lateral flagella have no effect on pathogenicity, but the polar flagellum is important in the pathogenesis of *V. parahaemolyticus* [36]. Therefore, we can suppose that the polar flagellum of NCCP15738 is the major machinery for swarming and has a pathogenic effect. In contrast, the lateral flagellum of NCCP15738 is likely related only to locomotion in this strain.

### Virulence factors

Even though NCCP15738 belongs to serotype O157, it causes diarrhea but not HUS in human hosts. Because of this, we were particularly interested in identifying the potential virulence factors within the genome of NCCP15738. The features that we identified through sequence analysis are detailed in Table 1, which includes a variety of pilus and fimbriae genes and their associated operons. However, NCCP15738 produces no shiga toxins, such as Stx1 (*stx1A*, *stx1B*) or Stx2 (*stx2A*, *stx2B*), and has only one locus of enterocyte effacement (LEE) that encodes type three secretion system (TTSS) (*escR*) [18]. From our comparison of NCCP15738 with *E. coli* K-12 substr. MG1655 and *E. coli* O157:H7 str. EDL933, we found that NCCP15738 has only one unique virulence gene, papD. NCCP15738 has 19 virulence genes and 18 of these genes had been previously reported in the other two strains.

**Table 1:** Virulence genes of NCCP15738

Category	Subcategory	Genes	Gene name
Adherence	*E. coli* common pilus (ECP)	fig|562.1020.peg.2959	ecpA
Adherence	*E. coli* common pilus (ECP)	fig|562.1020.peg.2960	ecpB
Adherence	*E. coli* common pilus (ECP)	fig|562.1020.peg.2961	ecpC
Adherence	*E. coli* common pilus (ECP)	fig|562.1020.peg.2962	ecpD
Adherence	*E. coli* common pilus (ECP)	fig|562.1020.peg.2963	ecpE
Adherence	*E. coli* common pilus (ECP)	fig|562.1020.peg.2958	ecpR
Adherence	F1C fimbriae	fig|562.1020.peg.2245	focC
Adherence	Type I fimbriae	fig|562.1020.peg.1416	fimA
Adherence	Type I fimbriae	fig|562.1020.peg.2244	fimA
Adherence	Type I fimbriae	fig|562.1020.peg.1417	fimB
Adherence	Type I fimbriae	fig|562.1020.peg.1414	fimC
Adherence	Type I fimbriae	fig|562.1020.peg.944	fimC
Adherence	Type I fimbriae	fig|562.1020.peg.1413	fimD
Adherence	Type I fimbriae	fig|562.1020.peg.2246	fimD
Adherence	Type I fimbriae	fig|562.1020.peg.3956	fimD
Adherence	Type I fimbriae	fig|562.1020.peg.4109	fimD
Adherence	Type I fimbriae	fig|562.1020.peg.1412	fimF
Adherence	Type I fimbriae	fig|562.1020.peg.2247	fimF
Adherence	Type I fimbriae	fig|562.1020.peg.1411	fimG
Adherence	Type I fimbriae	fig|562.1020.peg.2248	fimG
Adherence	Type I fimbriae	fig|562.1020.peg.1410	fimH
Adherence	Type I fimbriae	fig|562.1020.peg.2249	fimH
Adherence	Type I fimbriae	fig|562.1020.peg.1415	fimI
Auto transporter	Adhesin involved in diffuse adherence	fig|562.1020.peg.1313	aida
Iron uptake	Salmochelin siderophore	fig|562.1020.peg.1061	iroN
Secretion system	LEE locus encoded TTSS	fig|562.1020.peg.3365	escR

## Future directions

This study shows a broad comparative genomics approach to the study of the NCCP15738 genome and describes the features of this type of non-STEC O157. This information will be useful for studying the evolution of the pathogenic mechanisms in this strain and its adaptation to the environment.

## Availability of supporting data

Nucleotide sequence accession numbers: This Whole Genome Shotgun project has been deposited in DDBJ/EMBL/GenBank under the accession number ASHB00000000.

## Additional files

10.1186/s13099-016-0096-2 Bacterial strain list used for phylogenetic analysis.
10.1186/s13099-016-0096-2 Annotated genes of non-STEC O157 NCCP15738 using RAST server.
10.1186/s13099-016-0096-2 Lateral flagella genes of non-STEC O157 NCCP15738.

## Abbreviations

CDS: coding DNA sequences
EHEC: enterohemorrhagic *Escherichia coli*
GTR: generalized time-reversible
HGT: horizontal gene transfer
HUS: hemolytic-uremic syndrome
IS: insertion sequences
MLSA: multilocus sequence analysis
MLST: multi locus sequence typing
NCCP: National Culture Collection for Pathogens
ORFs: open reading frames
RAST: Rapid Annotation Using Subsystem Technology
STEC: shiga toxin-producing *Escherichia coli*
str.: strain
substr: substrain
